# The Hospitals Association

**Published:** 1893-08-12

**Authors:** 


### HOSPITAL MEETINGS.

### THE HOSPITALS ASSOCIATION.

It is not surprising in all the circumstances that a cheerful mood prevailed at the ninth annual meeting of the Association, held at St. Thomas's the other day. In the absence through illness of the President, Dr. Bristowe, Mr. Burdett took the chair, and moved the adoption of the report of the Council, read by Mr. Mitchelli, and of the Street Ambulance branch, read by Mr. Ryan. He called attention to the accounts which had been audited by Mr. Whinney, public accountant, which were laid on the table. Mr. Burdett congratulated the members present upon the very happy condition of the Hospitals Association, and upon the remarkable progress in sound principles of hos ital administration that had been made during the past ten years. So remarkable had this progress been that the Association had now arrived at a stage at which there was little to desire. Apart from all questions of the desirability of a Central Hospitals Board for London, it could certainly be said that such a board was not now so much needed as it was ten years ago. Hospitals had been recently having their share of attention from the press. The gooseberry season was with us, and editors —especially editors of evening newspapers—were in need of copy. After all, editors were but men, and one must sympathise with them in their difficulties. He (Mr. Burdett) had been accused of making a terribly radical speech, because he had pointed out at the meeting of the Council of the Metropolitan Hospital Sunday Fund that while the contributions from churches and chapels in the East End had this year increased, the contributions from the West End had decreased; but that was merely a plain statement of fact, the effect of which should certainly be to inspire the rich to do better. Speaking of the Street Ambulance branch, Mr. Burdett stated that when the Street Ambulance Association began its work the only means available in London for conveying the injured to hospital were the police ambulance and the cab. The police ambulance was not suited for the injured or sick, and the jolting of the cab frequently did much harm. Thanks to the Street Ambulance Branch and to the co-operation of Mr. Bischoffsheim and Mr. Ryan, all this had been changed, and there was now in London as complete a system of street ambulance as existed in any large city—as complete a system as could be desired. Mr. Burdett paid a warm tribute of personal affection and esteem to the late Dr. C. J. Steele in supplement to the reference to Dr. Steele's invaluable services to the association and the cause of hospitals contained in the report. Mr. Burdett had been associated with Dr. Steele in hospital work for a quarter of a century, and to him his death had been as the loss of one of his own family. Throughout his career Dr. Steele had been fearless in the advocacy of what he believed to be right. The aim of his whole life had been to make the hospital system of this country the most efficient in the world.

Mr. Timothy Holmes, F.R.C.S., seconded the adoption of the reports. He promulgated the view that it was very desirable to have an early decision as to whether a Central Hospital's Board was to be formed, because the proposal to form such a board was, if not the only, at least the most important result of the Lords' Committee. His own opinion was that such a board, if judiciously constituted, and its functions judiciously defined, or rather judiciously not defined, would be of the greatest service. If it were the case that a decision was delayed because Lord Sandhurst's time was fully taken up with political duties, then, said Mr. Holmes, pointedly, that made another reason why Lord Sandhurst should be relieved of his political duties as soon as possible. The hospitals of London were in a most chaotic condition. Some of them, such as St. Thomas's, where they were met, were the admiration of the world. Mr. Holmes spoke in high terms of the good work done by the Ambulance branch. He recommended the Ambulance Committee to take into consideration the formation of hospital stations, at which accidents might be treated. This might help to obviate the evils that were occasioned by the very unequal distribution of hospitals in London. If an accident occurred in a part of London remote from a hospital, the only place to which the patient could be taken was the police station.

The reports having been adopted, Rev. J. Marshall moved a vote of thanks to the Chairman, whose services to the hospital cause he warmly eulogised. Mr. Mitchelli seconded. Mr. Burdett moved votes of thanks to Mr. Mitchelli and Mr. Ryan. Mr. Mitchelli deserved the gratitude of the association for the generous way in which he devoted time to its service, notwithstanding that as Secretary of the Seamen's Hospital he was so fully occupied, and of

Mr. Ryan's enthusiasm in the cause of ambulance there could be no better proof than that he was going to devote Bank Holiday, as he always did, to inspecting ambulance stations. The officers and Council were then elected, Dr. Bristowe, who had again consented to accept office, being re-elected President.

